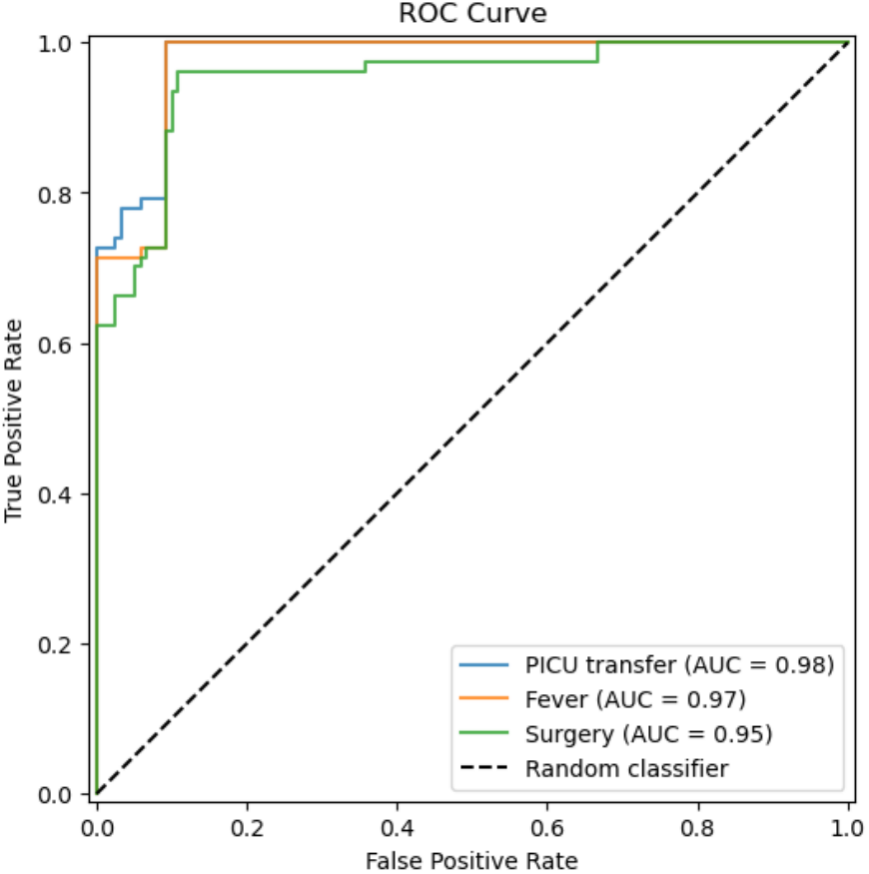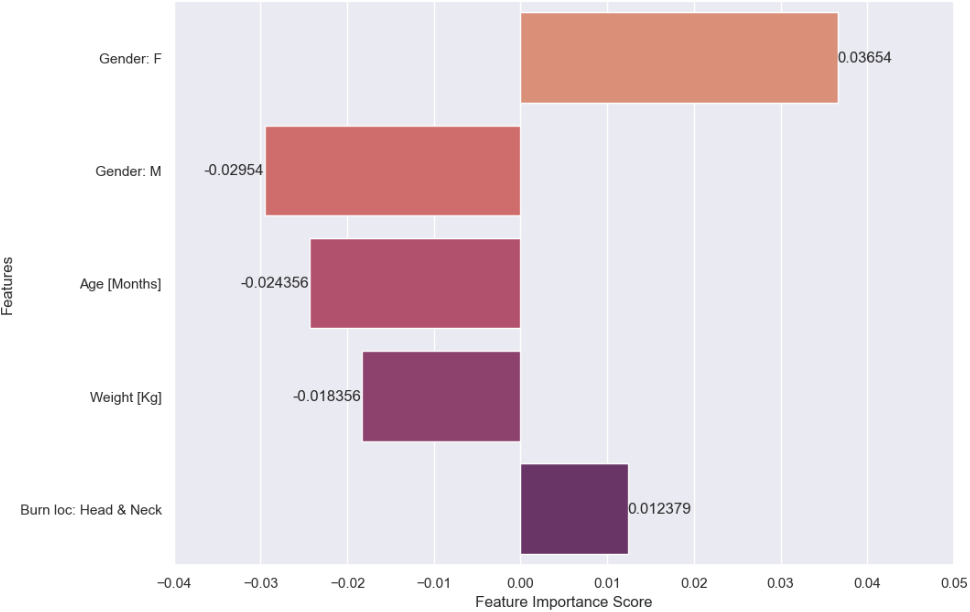# 48 Machine Learning-Based Predicting Model for Fever and Complications in Hospitalized Pediatric Burn Patients

**DOI:** 10.1093/jbcr/iraf019.048

**Published:** 2025-04-01

**Authors:** Lior Har-Shai, Sapir Gershov, Tomer Lagziel, Noa Manasseh, Alon Keller, Garry Tzarfati, Daniel Hilewitz, Dean Ad-El, Eyal Kalish, Asaf Olshinka

**Affiliations:** University of Texas Southwestern; Technion Institute of Technology; Icahn School of Medicine at Mount Sinai; Tel-Aviv University, Faculty of Medicine; Department of Plastic Surgery and Burns, Rabin Medical Center; Tel-Aviv University, Faculty of Medicine; Tel-Aviv University, Faculty of Medicine; Department of Plastic Surgery and Burns, Rabin Medical Center; Department of Plastic Surgery and Burns, Rabin Medical Center; Department of Plastic Surgery and Burns, Rabin Medical Center

## Abstract

**Introduction:**

Pediatric burn patients face significant risks of fever and complications, impacting morbidity, mortality, and healthcare costs. Despite advancements in burn care, early identification and management of these risks remain challenging. This study aimed to develop and validate a Machine Learning (ML) based predictive model using Random Forest (RF) algorithms to forecast the likelihood of fever and related complications in hospitalized pediatric burn patients.

**Methods:**

We conducted a retrospective analysis of pediatric burn patients admitted to a tertiary care center between 2012 and 2022. Data on patient demographics, burn severity, treatment interventions, and outcomes were extracted from medical records. RF models were trained and tested on this dataset to predict fever, necessity for pediatric intensive care unit (PICU) transfer, and surgical intervention requirements.

**Results:**

The allocated data contains 595 subjects, with an average age of 4.27±4.46 years and a Percent Total Body Surface Area (%TBSA) of 5.49±4.93. The RF models demonstrated high accuracy in predicting fever (F:1=0.82±0.028), ICU transfers (F:1=0.88±0.090), and the need for surgical interventions (F:1=0.81±0.027). Receiver operating characteristic (ROC) curve, comparing the performance of the predictive models for PICU transfer, fever, and surgery, demonstrated area under the curve (AUC) values of 0.98, 0.97 and 0.95 respectively. The feature importance analysis concluded that subjects who are females, young, low body weight, and with head and neck burn injuries are more likely to develop fever, undergo surgery, and be transferred to the PICU.

**Conclusions:**

Our Machine Learning-based predictive models show promise in identifying pediatric burn patients at increased risk for fever and complications, offering the potential for early intervention strategies. Future work will focus on prospective validation and integration into clinical workflows to enhance patient outcomes.

**Applicability of Research to Practice:**

By enabling early detection, optimizing resource allocation, facilitating personalized care, and reducing healthcare costs, these models have the potential to significantly enhance the management and outcomes of pediatric burn patients. The next steps involve validating these models in real-world settings and integrating them seamlessly into clinical workflows to fully realize their potential in improving pediatric burn care.

**Funding for the Study:**

N/A